# Supplementary material for: Diet quantity influences caste determination in honeybees (Apis mellifera)
Source: Proc Biol Sci. 2020 May 27;287(1927):20200614. doi: 10.1098/rspb.2020.0614 (PMC7287363; doi:10.1098/rspb.2020.0614)
Supplement: Supplemental Methods, Figures and Tables [file rspb20200614supp1.docx]

**Supplemental Methods**

***Determination of protein, carbohydrate, lipid and water contents in artificial diets***

Protein content of royal jelly was measured using a standard colorimetric Bradford assay (Sigma-Aldrich, MO, USA). Protein standards were prepared by diluting 1μl, 2.5μl, and 5μl of a stock albumin solution (200mg/ml) diluting in 1 ml of distilled water. The royal jelly test sample was prepared by diluting 1ml of the royal jelly in 10 ml of water. Five μl aliquots of each standard and the royal jelly sample were plated in a 96-well culture plate (Falcon, Corning, Durham, NC). The Bradford reagent (250μl) was then added to each well and the plates were left to develop at room temperature for 20 minutes. The samples absorbance at 595nm was measured using a spectrophotometer (Thermo Fischer Scientific, MA, USA).

Carbohydrate, lipid and water contents of royal jelly were calculated using a differential scanning calorimeter (DSC) (Perkin Elmer DSC Pyris 1, Waltham, MA, USA) by sealing 1 to 5 mg of royal jelly into a Perkin-Elmer aluminum DSC pan. An empty aluminum pan served as the control. The sample chamber was perfused with helium gas at 10 ml/min during the scan. The temperature was ramped down from 25°C to -100°C at a rate of 1°C /min using a liquid cooling accessory (Perkin Elmer Cryofill, Waltham, MA, USA) to determine both freezing and melting characteristics. Water melts near 0°C, generating an endotherm peak in the calorimetric scan, and the sugars caused distinct glass transitions between -20°C and -40°C. The area of the peaks generated during the events were calculated and used to determine the water content. The freezing point depression was measured in royal jelly using a calibration based on glucose and fructose solutions in deionized water (5:7%, 7:10%, and 10:10% of glucose: fructose) (40). In an Eppendorf tube, 100 μl of Royal jelly was diluted in 900 μl of 100% ethanol, vortexed for 1 minute, and chilled for 2 hours at -80°C. Then the tubes were centrifuged at 14000 rpm for 30 minutes. The supernatant was removed, dried in flowing nitrogen gas, and reconstituted in 1ml of deionized water. Five μl of this solution was analyzed in the DSC in five replicates, and the freezing and melting points were noted and compared against glucose and fructose standards to obtain an estimate of the sugar content in royal jelly.

**Supplemental Table**

**Table S1. Survival by diet treatment.** For each treatment, the top value is the number of first instar larvae put into treatment, the second is the number of adults that eclosed, and the third is the percent survival. The *ad libitum* treatment was reared on the medium-protein, medium-carbohydrate diet.

**Table S2.** Number of individuals found in each hierarchical cluster determined by ward linkage organized by diet quality. Diets were high protein- high carbohydrate (HPHC), high protein- medium carbohydrate (HPMC), high protein- low carbohydrate (HPLC) etc. The *ad lib* treatment was included in the medium protein-medium carbohydrate treatment (MPMC) for this analysis.

| Cluster | Queen Control | HPHC | HPMC | HPLC | MPHC | MPMC* | MPLC | LPHC | LPMC | LPLC | Worker Control |
| --- | --- | --- | --- | --- | --- | --- | --- | --- | --- | --- | --- |
| 1 | 20 | 12 | 13 | 9 | 10 | 27 | 10 | 0 | 0 | 0 | 0 |
| 2 | 0 | 23 | 20 | 15 | 30 | 24 | 13 | 2 | 0 | 1 | 0 |
| 3 | 0 | 20 | 10 | 5 | 14 | 19 | 6 | 1 | 0 | 0 | 43 |
| In queen cluster | | 22% | 30% | 31% | 19% | 39% | 34% | 0% | 0% | 0% |  |

*Includes the *ad lib* feeding treatment

**Supplemental Figures**

**
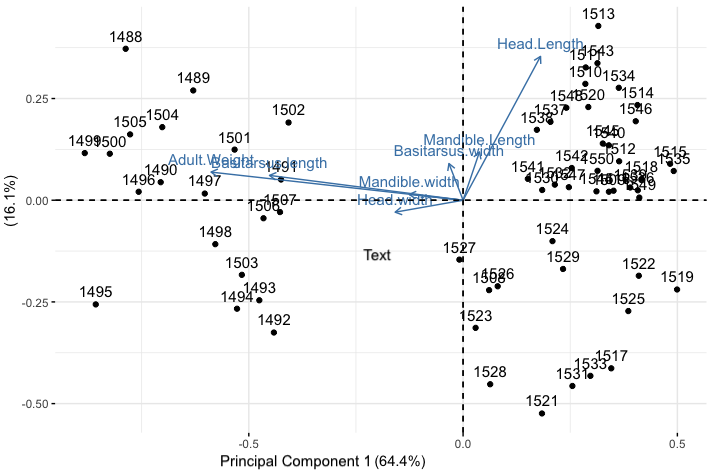
**

**Figure S1.** Vectors for each of the variables included in the principal component analysis. The principle component analysis was conducted on commercially-reared queens (20 individuals, numbers 1488-1507) and hive-reared workers (40 individuals, numbers 1508-1550). Based on these loadings, values for PC1 and PC2 were calculated for the in vitro reared individuals.

**
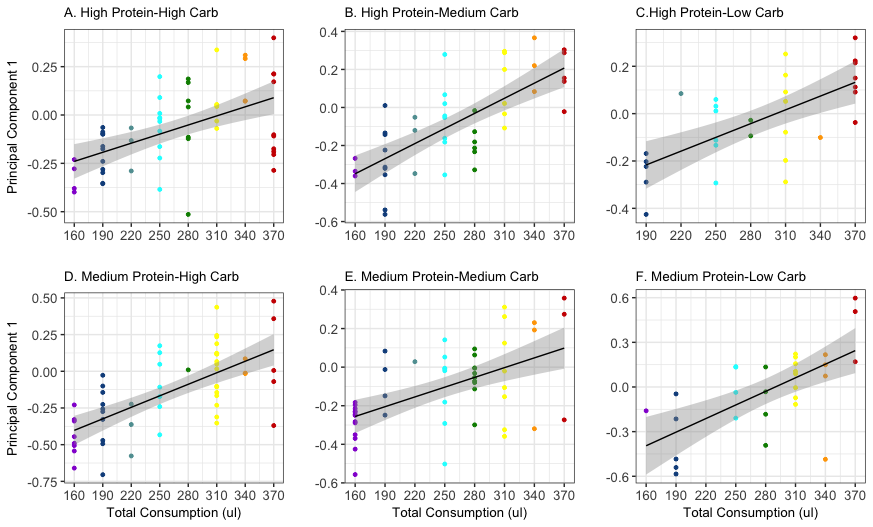
**

**Figure S2.** Effect of diet quantity on PC1 for six of the nine artificial diets (A-F). Low protein diets were excluded because these treatments had a high mortality. (A) High Protein-High Carbohydrates (P<0.0001, R^2^= 0.2657), (B) High Protein-Medium Carbohydrate (P<0.0001, R^2^= 0.512), (C) High Protein-Low Carbohydrate (P<0.0001, R^2^= 0.418), (D) Medium Protein-High Carbohydrate (P<0.001, R^2^= 0.512), (E) Medium Protein- Medium Carbohydrate (P<0.0001, R^2^= 0.271), (F) Medium Protein-Low Carbohydrate (P=0.002, R^2^= 0.395).
